# Recent advances in rehabilitation for Parkinson’s Disease with Exergames: A Systematic Review

**DOI:** 10.1186/s12984-019-0492-1

**Published:** 2019-01-29

**Authors:** Augusto Garcia-Agundez, Ann-Kristin Folkerts, Robert Konrad, Polona Caserman, Thomas Tregel, Mareike Goosses, Stefan Göbel, Elke Kalbe

**Affiliations:** 1 Multimedia Communications Lab, Technische Universitaet Darmstadt, Darmstadt, Germany; 2 Department of Medical Psychology | Neuropsychology and Gender Studies & Center for Neuropsychological Diagnostics and Intervention (CeNDI), University Hospital Cologne, Cologne, Germany

**Keywords:** Parkinson’s Disease, Cognitive Impairment, Rehabilitation, Cognitive training, Exergames, Serious Games

## Abstract

**Objective:**

The goal of this contribution is to gather and to critically analyze recent evidence regarding the potential of exergaming for Parkinson’s disease (PD) rehabilitation and to provide an up-to-date analysis of the current state of studies on exergame-based therapy in PD patients.

**Methods:**

We performed our search based on the conclusions of a previous systematic review published in 2014. Inclusion criteria were articles published in the indexed databases Pubmed, Scopus, Sciencedirect, IEEE and Cochrane published since January 1, 2014. Exclusion criteria were papers with a target group other than PD patients exclusively, or contributions not based on exergames. Sixty-four publications out of 525 matches were selected.

**Results:**

The analysis of the 64 selected publications confirmed the putative improvement in motor skills suggested by the results of the previous review. The reliability and safety of both Microsoft Kinect and Wii Balance Board in the proposed scenarios was further confirmed by several recent studies. Clinical trials present better (*n* = 5) or similar (*n* = 3) results than control groups (traditional rehabilitation or regular exercise) in motor (TUG, BBS) and cognitive (attention, alertness, working memory, executive function), thus emphasizing the potential of exergames in PD. Pilot studies (*n* = 11) stated the safety and feasibility of both Microsoft Kinect and Wii Balance Board, potentially in home scenarios as well. Technical papers (*n* = 30) stated the reliability of balance and gait data captured by both devices. Related meta-analyses and systematic reviews (*n* = 15) further support these statements, generally citing the need for adaptation to patient’s skills and new input devices and sensors as identified gaps.

**Conclusion:**

Recent evidence indicates exergame-based therapy has been widely proven to be feasible, safe, and at least as effective as traditional PD rehabilitation. Further insight into new sensors, best practices and different cognitive stadiums of PD (such as PD with Mild Cognitive Impairment), as well as task specificity, are required. Also, studies linking game parameters and results with traditional assessment methods, such as UPDRS scores, are required. Outcomes for randomized controlled trials (RCTs) should be standardized, and follow-up studies are required, particularly for motor outcomes.

**Electronic supplementary material:**

The online version of this article (10.1186/s12984-019-0492-1) contains supplementary material, which is available to authorized users.

## Introduction

Parkinson’s Disease (PD) is caused by the progressive degeneration of dopaminergic neurons in the *substantia nigra pars compacta*, reduced striatal dopamine, and the presence of Lewy Bodies. Its most common form, Idiopathic Parkinson’s Disease, is determined by cardinal symptoms: Rest tremor, asymmetry, bradykinesia and a good response to dopamine confirm the pathology in 99% of cases.

Besides motor symptoms, cognitive dysfunctions occur very often in PD Patients [1, 2]. Once cognitive impairment can be objectified in PD patients, it is called Mild Cognitive Impairment in PD (PD-MCI). Criteria for a diagnosis of PD-MCI following the Petersen criteria includes a gradual decline in cognitive ability in the context of PD, cognitive deficits on a formal neuropsychological test and a cognitive deficit which is not sufficient to interfere with functional independence, but presenting subtle difficulties on complex functional tasks [3]. The prevalence of PD-MCI among PD patients is around 25% [4]. Furthermore, PD-MCI is a risk factor for a further cognitive decline into PD Dementia (PDD), which is characterized by cognitive symptoms in at least two domains (e.g. memory, executive function) which cause dysfunction in activities of daily living (ADL). PDD is an exclusion criterion for some PD treatments [5] such as Deep Brain Stimulation, and there is no strategy to prevent cognitive decline in PD patients or approved pharmacological approach to treat PD-MCI at the moment [5]. However, recent research suggests that cognitive function can be improved, or stabilized, in patients with PD through cognitive training [6–8]. Although at this point the ideal type or intervention frequency of cognitive training therapy for PD patients with either PD-MCI or initial PDD is not clear, several clinical studies indicate cognitive training improves executive and memory functions in PD patients. Meta-analyses also stress the need to provide clear inclusion and exclusion criteria in interventions for cognitive training and PD, considering the three stages of cognitive decline in PD [9]. It is also discussed that a combined treatment of cognitive and physical training seems to be a good option [10, 11]. Additional advantages of this strategy are transfer effects between cognitive and motor skills, for example, the positive effects of cognitive training in physical symptoms such as freezing of gait [12].

It is precisely due to the potential transfer effects that exergames [13] show great potential for PD rehabilitation since one of the best methods to combine both cognitive and physical training is the use of video games which require the user to perform physical movements while conducting cognitive exercises. Exergames, a portmanteau of exercise and games, aim to combine the motivational aspects of playing with the physical benefits of exercise. In regard to target groups and activities, such as exergames for rehabilitation, adapting to the particular needs of the users, and to their physical and cognitive capabilities, are usually mentioned as significant advantages over alternative rehabilitation methods [14]. Additionally, the confirmed reliability of sensory feedback from traditional exergaming platforms such as the Wii Balance Board (WBB) or Microsoft Kinect is backed by numerous publications from recent years [15–33].

On the specific topic of exergames for PD rehabilitation, a systematic review published in 2014 by Barry et al. [34] found a total of 1121 abstracts related, to some degree, to the use of exergames in PD. Seven clinical studies out of those publications were selected and analyzed. Their conclusions showed both, the promising future of exergame-based PD therapy, as well as the lack of rigorous clinical studies to evaluate its effectiveness. Research in this area in recent years has been extensive, but to this point, recent meta-analyses do not cover the advance in the specific research area of exergame-based PD rehabilitation. The goal of the present systematic review is thus to study the progress of this area during the years 2014 to 2017, with its focus set on sensors and controllers, specifically comparing sensors based on RCT outcomes where possible, as well as proposing the use of additional sensors where available, thus providing an up-to-date critical view of the work performed so far, and identifying the remaining steps required until exergame-based therapy may become a clinical standard for PD patients.

## Methods

In order to update the previous systematic review [34], which covered publications until December 2013, we initially searched for publications using Barry et al’s previous searchline:

Exergam* OR active video gaming OR Microsoft Kinect OR Kinect OR Nintendo Wii OR Wii OR Sony EyeToy OR IREX OR Dance Dance Revolution AND Parkinson*.

However, we found that Barry’s searchline excluded significant research, and we thus updated the query „active video gaming “ to the more comprehensive „video gam* “ instead. We also removed duplicates, with a final result of:

Exergam* OR Video Gam* OR Kinect OR Wii OR Sony EyeToy OR IREX OR Dance Dance Revolution AND Parkinson*.

**Fig. 1:**
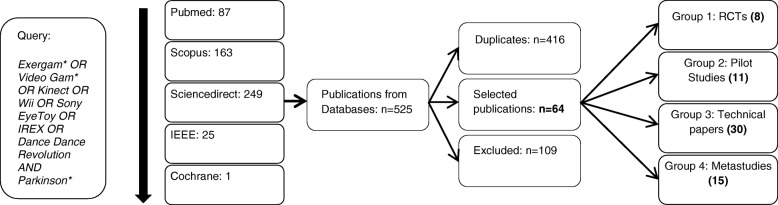
Flow Diagram of the study selection process

Inclusion criteria were articles published in the indexed databases Pubmed, Scopus, Sciencedirect, the Institute of Electrical and Electronics Engineers (IEEE) and Cochrane published since January 1, 2014. Publications were not included for duplicates or if the topic did not relate to this review despite a string match, that is, if (A) the target group was not PD patients exclusively, or (B) the employed therapy was not based on exergames. Given the variety of outcomes of publications regarding exergame therapy for PD, and considering most studies provide two or three outcomes, we limited our analysis to the main three outcomes given in case more than three were provided. In case we had to limit the outcomes of a study, standard ones were chosen where possible. The search was performed on February 26, 2018, and again on November 27, 2018. The methods are presented in the flow diagram shown in Fig. 1. This yielded the following results, excluding the aforementioned systematic review and the randomized clinical trials (RCT) identified in it, which were nevertheless also included in our analysis:

- Pubmed: 87 matches, of which we excluded 36 papers, leaving us with 51 results.
- Scopus: 163 matches, 8 Additional non-duplicate publications using the same criteria.
- Sciencedirect: 249 matches, 5 Additional non-duplicate publications using the same criteria.
- IEEE: 25 matches, 0 further results.
- Cochrane Library: 1 match, 0 further results.

From the 109 non-duplicate excluded papers, 77 were excluded due to criterion A and 32 due to criterion B. This reduced our corpus to a total of 64 publications, which we then proceeded to classify into the following groups:

- Group 1. RCTs of game- or game-technology based methods in PD therapy. These are the primary focus of this paper.
- Group 2. Pilot studies of game- or game-technology based methods in PD therapy (non-RCTs). These are the secondary focus of this paper.
- Group 3. Technical papers, with no specific therapeutic focus (for example reliability tests). The goal here is to provide an overview on recent technical advances in the sector. This is summarized in the discussion section.
- Group 4. Systematic reviews and meta-analyses relating to the topic. The contents and goals of these reviews are summarized in the discussion section.

In order for a study to qualify as an RCT, we used the question and scoring criteria presented in Barry et al’s systematic review [34], in which a study has RCT consideration if satisfactorily answers the following questions:

- Is inclusion and exclusion criteria stated?
- Are participant characteristics described in detail? (number, age, sex, disease severity)
- Is sample size justified?
- Is group randomization explained?
- Is the design clear?
- Were exergaming sessions explained in detail?
- Were baseline and post-test data collected?

We also designated a publication as an RCT if all of these questions were satisfactorily answered. We also decided to include the previously existing RCT [35], for comparison.

## Results

As a first step, we studied the input devices used on each publication in groups 1 to 3. The results are presented in Table 1, while technical data about the sensors mentioned in this publication is presented in Table 7. We noticed there is a strong focus on using the Microsoft Kinect for PD rehabilitation (36 of 49 publications used the Kinect) in comparison with other approaches, such as the WBB, custom sensors or, in two cases, a Wii hand controller (WiiMote). RCTs seem to focus on the Kinect as well (3 out of 8 studies).

**Table 1:** Input devices used in the analyzed publications. A pilot study [54] used both the Kinect and Wii sensors, and thus represented in parenthesis in both columns. For the total count it is considered as Kinect

	Kinect	WBB	WiiMote	Custom Device/Other	Total
Group 1: RCT	3	2	1	2	8
Group 2: Pilot	8(9)	2(3)	0	0	11
Group 3: Technical	24	3	1	2	30
Total	**36**	**7**	**2**	**4**	**49**

### Randomized Clinical Trials (RCTs)

The quality of studies in the area has drastically improved in the last 4 years, and 8 new RCTs analyzing the use of exergames in PD rehabilitation were identified in this review [36–43]. Therefore, from this point onwards we refer to a total of 9 RCTs including the one identified in the previous review. A comprehensive list of the identified RCTs is presented in Additional file 1.

Given the variety of outcomes of these works, we limited this section to the main three outcomes. Standard outcomes, such as UPDRS Scores, MOCA Scores or standard tests (Berg Balance Scale (BBS), Timed Up and Go Test (TUG) were chosen where possible. Information about the cognitive and motor dysfunctions of the control and intervention groups can be extrapolated from pre-test UPDRS and MMSE scores, which are provided where available. Tables 2 and 3 present a summary of all RCTs, particularly participants, aim, duration and type of exergame, condition trained and methods, settings, outcomes, and conclusions. Table 4 compares RCT outcomes where possible. All studies had active control groups, and present results equal or better than this control groups (which performed traditional rehabilitation therapy or regular exercise), although at this point it is still difficult to determine which therapy works best. We consider the results of Allen et al. [36], and Song et al. [41] to be of particular interest since the outcomes of the intervention and control groups were similar although the evaluation was performed at home in a minimally supervised scenario. Both authors insist on the need to consider task specificity in the future.

**Table 2:** RCT Experiment Settings. *ADL* = Activities of Daily Living, *BBS* = Berg Balance Scale, *C* = Control Group, *H&Y* = Hoehn and Yahr Scale, *I* = Intervention Group, *MMSE* = Mini Mental Scale Examination, *PD* = Parkinsons Disease, *UPDRS* = Universal Parkinsons Disease Rating Scale, *WBB* = Wii Balance Board

Author	Participants: n, age (C/I),sex (men C/I),duration (y), MDS-UPDRS (C/I))	Inclusion Criteria	Exclusion Criteria	Aim	Sensor / Platform	Game	Intervention	Control	Setting
Pompeu et al. (2012) [35]	N: 32 Age (y): 67.4 Sex (men): 17 Duration (y): MDS-UPDRS: 8.9/10.1	• Diagnosis of idiopathic PD • Age 60–85 • H&Y Stages to II • Good visual and auditory acuity • 5 to 15 years of education	• Other neurological or orthopaedic diseases • MMSE< 23 • Depression (GDS < 6)	Evaluate Wii-based motor cognitive training versus traditional balance exercises in UPDRS ADL scores	WBB	Nintendo Wii Fit	Fourteen sessions of 30 min of stretching, strengthening and axial mobility exercises, plus 30 min of Wii Fit training, two sessions per week for 7 weeks Total time: 14 h	Fourteen sessions of 30 min of stretching, strengthening and axial mobility exercises, plus 30 min of balance therapy without cognitive stimulation. Two sessions per week for 7 weeks	Clinical, with supervision of a physiotherapist
Allen et al. (2017) [36]	N: 38 Age (y): 67.5/68.4 Sex (men): 12/11 Duration (y): 7.9/8.7 MDS-UPDRS: 41.3/38.8 (Motor)	• Diagnosis of idiopathic PD • Age > =40 • Stable medication	• Upper Extremity Pain or injuries • MMSE < 24 • Any health condition that would interfere with safe conduct	Evaluate upper extremity exergames to improve arm and hand activity	Custom developed sensor, Tablet	Custom developed games	Around 30 min per session, 3 times per week for 12 weeks Total time: 18 h	Usual care and activities	At home, unsupervised
Liao et al. (2015) [37]	N: 36 Age (y): 64.6/65.1/67.3 (2 Intervention groups) Sex (men): 5/6/6 Duration (y): 6.4/6.9/7.9 MDS-UPDRS:Not provided	• Diagnosis of Idiopathic PD • H&Y Scale I to III • Ability to walk unaided • Stable medication	• MMSE < 24 • Unstable medical condition • Other neurological, cardiopulmonary or orthopedic diseases • Past history of seizure • Pacemaker • Vision deficits	Evaluate exergames on obstacle crossing performance and dynamic balance	WBB	Wii Fit Plus	12 sessions, 2 per week, over a 6-week period 45 min Wii Fit plus 15 min treadmill TE Group: 60 min treadmill Total time: 12 h	Fall prevention education, and regular exercise	Clinical, with supervision of a physiotherapist
Shih et al. (2016) [38]	N: 22 Age (y): 67.5/68.8 Sex (men): 9/7 Duration (y): 4.03/5.22 MDS-UPDRS: Not provided MMSE: 27.4/28.2	• Diagnosis of idiopathic PD • H & Y Scale I to III • Stable medication • Standing unaided	• MMSE < 24 • Other neurological, cardiovascular or orthopedic diseases • Uncontrolled chronic diseases	Evaluate a therapeutic exergames based on Kinect, compared to traditional balance training	Kinect	Custom developed games	30 min balance-based exergaming (reaching fixed object, moving object, obstacle avoidance and marching) + Balance training, 50 min per session, 2 sessions/week, 8 weeks Total time: 13.3 h	30 min conventional balance training+ Balance training, 50 min per session, 2 sessions/week, 8 weeks	Clinical, supervision not specified
Ribas et al. (2017) [39]	N: 20 Age (y): 61.7/60.2 Sex (men): 4/4 Duration (y): 6.5/7 MDS-UPDRS: 22.5/20.5 MMSE: 27.5/27.5	• Diagnosis of idiopathic PD • Age 40–80 • H & Y Scale I to III • BBS > 45	• MMSE < 24 • Acute pain, comorbid conditions • Visual Impairment or assistive devices • Previous experience with WBB • Receiving any other therapy	Determine the effectiveness of WBB exergaming in improving balance, fatigue, capability and quality of life	WBB	Custom developed games	30 min exergaming with prior practicing of the required postures and movements. 2 sessions per week for 12 weeks Total time: 12 h	Warming, stretching, and active exercises (10 min) resistance for limbs (10) and diagonal exercises for trunk neck and limb (10). 12 weeks of sessions twice a week	Clinical, supervised by two physiotherapists
Zimmermann et al. (2014) [40]	N: 39 Age (y): 69.9/66.3 Sex (men): 13/12 Duration (y): 5.1/5.2 MDS-UPDRS: 24/25 MMSE: 28/28.75	• Diagnosis of idiopathic PD	• Moderate or severe demented • Other neurologic conditions • Insufficient knowledge of German	Compare a cognition-specific computer-based cognitive training program with a motion-controlled computer sports game for effects in cognitive performance	Wiimote	Wii Sports Resort	4 tasks, 10 min per task, 3 times per week for 4 weeks with Wii: Table tennis, swordplay, archery and air sports. Total time: 8 h	4 tasks, 10 min per task, 3 times per week for 4 weeks with Cogniplus: Focused attention, working memory, executive function and response inhibition	Clinically supervised by a psychologist or trained student
Song et al. (2017) [41]	N: 60 Age (y): 65/68 Sex (men): 9/15 Duration (y): 9/7 MDS-UPDRS: 33/31 (Motor) MMSE: 29/28	• Diagnosis of idiopathic PD • Age > =40 • Able to walk unaided 30 m • Stable medication	• MMSE < 24 • Other medical conditions	Determine the efficacy of a home-based rehabilitation scenario	Custom sensor (Dance mat)	Modified version of Stepmania	Minimum of 15 min per session, three sessions per week for 12 weeks. Difficulty adapted to patient progress manually. Total time: 9 h	Usual healthcare	At home, minimally supervised (initial instruction and visits)
Ferraz et al. (2018) [42]	N: 62 Age (y): 71/67/67 (2 Intervention Groups) Sex (men): 18/11/10 Duration (y): 4/6/4 MDS-UPDRS: 29.86/28.65/32.30 MMSE: 27/27/27	• Diagnosis of idiopathic PD • Age > = 60 • H & Y Scale II to III • Able to walk unaided • Stable medication	• Other medical conditions • Visual or hearing impairment • Alcohol or toxic substance use • Contraindications for sport • Practicing physical exercise programs recently	Compare functional training, bicycle exercise and Kinect exergaming	Kinect	Kinect Adventures	30 min per session plus stretching and breathing exercises, three sessions per week for 8 weeks. Bicycle group: Same session length, max-HR-based training with increased demand for every week. Total time: 12 h	Functional training, 10 activities lasting 3 min each, three sessions per week for 8 weeks.	Clinically supervised by a physiotherapist
Tollar et al. (2018) [43]	N: 74 Age (y): 67.5/70.0/70.6 (2 Intervention Groups) Sex (men): 13/12/11 Duration (y): 7.3/7.5/7.5 MDS-UPDRS: 19.0/18.2/18.9 MMSE: Not Provided	• Diagnosis of idiopathic PD • H & Y Scale II to III • Stable medication • Mobility, Balance or Postural Problems	• MMSE < 24 • Beck Depression Score > 40 • Other diseases • Recent surgery • Use of DBS	Compare bicycle exercise and Kinect exergaming	Kinect	Kinect Adventures	60 min per session, five sessions per week for 5 weeks. Bicycle group: HR-controlled (110–140 bpm) spinning class Exergaming group: Reflex Ridge, Space Pop and Just Dance. Total time: 25 h	Usual healthcare	Clinically supervised by a physiotherapist

**Table 3:** RCT Experiment Outcomes. MOCA = Montreal Cognitive Assessment, PDQ-39 = Parkinsons Disease Questionnaire, TUG = Time Up and Go Test. Results are presented as mean (standard deviation) unless stated otherwise. Outcomes are adimensional where no units are stated

Author	Outcome and statistics	Baseline (Control mean (SD) /Intervention mean (SD))	Post Intervention (Control mean (SD) /Intervention mean (SD))	Follow up (Control mean (SD) /Intervention mean (SD))	Main Results	Main conclusion
Pompeu et al. (2012) [35]	• UPDRS-II Score (ADL) • BBS • MOCA Test (Cognition) • RM-ANOVA	• 8.9 (2.9) / 10.1 (3.8) • 51.9 (4.6) / 52.9 (4.1) • 21.7 (4.6) / 20.6 (4.5)	• 7.6 (2.9) / 8.1 (3.5) • 53.1 (3.4) / 54.4 (2.2) • 23.1 (4.6) / 22.2 (4.5)	• 2 months FU • 8.1 (3.2) / 8.3 (3.6) • 53.1 (3.1) / 54.1 (2.0) • 23.3 (3.4) / 21.8 (4.5)	Post-hoc Tukey tests comparing before and after training comparing control and intervention not statistically significant. No significant group differences between intervention and control groups	Exergames as effective as traditional balance therapy
Allen et al. (2017) [36]	• Nine hole peg test (s) • Horizontal Tapping test (taps/60s) • Horizontal Tapping test (error score) • ANCOVA	• 28.8 (5.7) / 29.9 (7.3) • 124.1 (34.9) / 119.0 (29.4) • 0.047 (0.064) / 0.048 (0.042)	• 29.0 (7.8) / 30.4 (7.5) • 130.1 (30.4) / 114.6 (26.3) • 0.070 (0.059) / 0.041 (0.037)		Two-sided t-test, intervention minus control values not statistically significant for nine hole peg test (*p* = 0.84), statistically significant for horizontal tapping test (*p* = 0.006) and error score (*p* = 0.02) No significant group differences between intervention and control groups in primary or secondary outcome. Improvements in some areas, decline on others	Exergames should consider task specificity
Liao et al. (2015) [37]	• Obstacle Crossing Performance speed (cm/s) • TUG (s) • PDQ-39 • ANOVA	• 80.4 (16.1) / 77.5 (21.8) / 75.2 (11.4) • 11.9 (2.7) / 12.1 (2.1) / 12.6 (4.1) • 78.2 (23.3) / 82.2 (27.3) / 84.5 (26.0)	• 78.5 (17.0) / 85.8 (18.0) / 87.0 (16.5) • 12.6 (3.6) / 11.0 (1.8) / 9.7 (2.1) • 79.0 (24.3) / 70.8 (27.1) / 68.8 (20.0)	• 1 month FU • 78.2 (17.3) / 84.7 (21.4) / 91.1 (20.0) • 12.9 (3.8) / 10.7 (1.5) / 9.7 (2.3) • 80.2 (24.5) / 70.0 (26.5) / 65.8 (18.3)	Statistically significant differences between intervention and control groups for obstacle crossing at post (*p* < 0.001) and follow-up (*p* < 0.001) Statistically significant differences between intervention and control groups for TUG at post (*p* < 0.001) and follow-up (*p* < 0.001) Statistically significant differences between intervention and control groups for PDQ-39 at post (*p* = 0.004) and follow-up (*p* = 0.001) but no differences between treadmill and Wii groups. Wii group shows greater improvement than the other intervention group and control group	Significant improvement of patients with Wii training
Shih et al. (2016) [38]	• BBS • TUG (s) • LOS Reaction time (s)	• 50.9 (5.32) / 50.4(4.79) • 9.5 (2.45) / 10.05 (4.66) • 0.96 (0.33) / 0.88 (0.24)	• 53.2 (2.86) / 53 (1.89) • 8.71 (1.8) / 9.18 (3.42) • 0.74 (0.24) / 0.79 (0.18)		t-test statistically significant for the control and intervention groups in BBS and TUG, and only in the intervention group for LOS. Intervention group shows better results than control group, both having positive effects.	Exergaming at least as effective as traditional balance therapy
Ribas et al. (2017) [39]	• BBS • Fatigue severity scale • 6MWT(m) • RM-ANOVA	• 48.4 (2.63) / 50.4 (2.79) • 3.55 (1.68) / 3.80 (1.66) • 384 (86.43) / 352 (91.99)	• 48.2 (2.89) / 52.3 (2.26) • 3.02 (1.22) / 1.83 (0.57) • 437 (89.69) / 408 (97.27)	• 2 months FU • 46.9 (2.72) / 47.7 (4.80) • 3.23 (1.31) / 3.05 (1.11) • 392 (80.24) / 376 (98.68)	Post-hoc Bonferroni tests before vs. after for intervention *p* = 0.033, for control *p* < 0.001, before vs. Follow-up *p* = 0.022 for intervention, *p* = 0.037 for control. Increase in balance for the intervention group, not sustained at follow-up. Significant improvement in fatigue for both groups, not sustained at follow-up	Exergames seem to empower motivation and achieve significant results in balance. Future studies should study the effect of exergames on fall risk.
Zimmermann et al. (2014) [40]	• Neurophysiological tests for alertness, working memory, executive function (Cognition) • MANOVA	• 272 / 291 (Median) • −0.16 / -0-05 (Median) • 2.3 / 2–17 (Median)	• 266 / 275 (Median) • − 0.14 / -0.16 (Median) • 2.44 / 2.37 (Median)		t-test statistically significant for alertness (*p* = 0.024) but not significant for working memory (*p* = 0.431) or executive function (*p* = 0.462). Better performance in attention in intervention than in control group	Non-cognitive-specific exergame therapy may deliver the same degree of cognitive benefit than cognition-specific computerized training
Song l et al. (2017) [41]	• Stepping performance CSRT • TUG (s) • MOCA Test (Cognition) • ANCOVA	• 847 (221) / 824(176) • 9.51 (2.27) / 9.57 (2.38) • 26.5 (2.70) / 26.4 (2.77)	• 794 (88) / 798 (169) • 9.02 (1.70) / 9.72 (2.14) • 26.7 (2.3) / 27.3 (2.8)		Difference between groups not statistically significant for CSRT (*p* = 0.59), and MOCA (*p* = 0.69). Difference statistically significant for TUG (*p* = 0.02). No effect in step performance, mobility, muscle power, cognition, reaction time, FOG, or falls	Task-specific training may be required. Sessions may need to be longer. Further research with severe PD patients required. Ensuring safety in a home scenario may hamper effective results.
Ferraz et al. (2018) [42]	• 6MWT (m) • 10MWT (s) • PDQ-39 • ANOVA	• 354.9 (98.9) / 405.2 (97.3) / 365.4 (81.1) • 1.3 (0.3) / 1.3 (0.3) / 1.2 (0.3) • 47 (25.1) / 38.1 (19.8) / 44.7 (26.7)		• 1 month FU • 391.7 (107.5) / 440.2 (90.2) / 401.2 (77.9) • 1.4 (0.4) / 1.4 (0.3) / 1.4 (0.3) • 41.7 (21.7) / 32.9 (19.1) / 33.9 (25.2)	t-tests pre- vs. post intervention statistically significant for control (*p* = 0.08), bicycle (*p* = 0.001) and exergaming (*p* = 0.005) groups for 6MWT. For 10MWT, not significant (*p* = 0.068, *p* = 0.101) for control and bicycle groups but significant (*p* = 0.011) in exergaming group. For PDQ-39 not significant (*p* = 0.069, *p* = 0.185) for control and bicycle groups but significant (*p* = 0.004) in exergame group. All groups show significant improvement in 6MWT, only exergame group shows significant improvement in 10MWT. PDQ improvement not significant.	Exergame training has similar outcomes to functional training and bicycle exercise, all therapies present improvements on walking capacity, ability to stand up, sit and functionality.
Tollar et al. (2018) [43]	• UPDRS-II • BBS • 6MWT (m) • ANOVA	• 19.0(4.67)/18.2(3.85)/18.9(3.11) • 26.3(5.21)/23.6(3.60)/22.7(4.24) • 270.2(90.66)/204.6(34.94)/222.4(40.85)	• 18.9(2.19)/13.7(2.45)/15.7(2.59) • 24.9(5.91)/32.4(4.61)/26.9(4.17) • 253.9(81.61)/334.2(68.90)/364(51.53)		Tukey’s post hoc contrast. Significant (*p* < 0.05) improvement in all outcomes except UPDRS, with no difference between exergame and cycling groups. Improvement in UPDRS is present (−4.5 points for exergame and − 3.2 for cycle) but not significant.	Exergame therapy has similar outcomes to bicycle training

**Table 4:** Comparison of RCT outcomes. Results are presented as mean (standard deviation) unless stated otherwise. Outcomes are adimensional where no units are stated

Outcome: TUG (s) (Lower is better)	Method	Control - Before	Intervention - Before	Control - After	Intervention - After	Difference control	Difference Intervention
Liao et al. (2015)	WBB - Wii Fit Plus	11.9 (2.7)	12.6 (4.1)	12.6 (3.6)	9.7 (2.1)	0.7	−2.9
Shih et al. (2016)	Kinect - Custom Game	9.5 (2.45)	10.05 (4.66)	8.71 (1.8)	9.18 (3.42)	−0.79	−0.87
Song et al. (2017)	Dance Mat – Stepmania	9.51 (2.27)	9.57 (2.38)	9.02 (1.70)	9.72 (2.14)	−0.49	0.15
Outcome: BBS (Higher is better)							
Pompeu et al. (2012)	WBB - Wii Fit	51.9 (4.6)	52.9 (4.1)	53.1 (3.4)	54.4 (2.2)	1.2	1.5
Shih et al. (2016)	Kinect - Custom Game	50.9 (5.32)	50.4 (4.79)	53.2 (2.86)	53 (1.89)	2.3	2.6
Ribas et al. (2017)	WBB - Custom Game	48.4 (2.63)	50.4 (2.79)	48.2 (2.89)	52.3 (2.26)	−0.2	1.9
Tollar et al. (2018) [43]	Kinect – Kinect Adventures	26.3 (5.21)	23.6 (3.60)	24.9 (5.91)	32.4 (4.61)	−1.4	8.8

We still observe several deficiencies in more recent RCTs, namely the lack of standardized outcomes, follow-up protocols and patient assessment methods, which are further described in the discussion of this paper. Regarding the risk of bias, seven of the clinical trials are single-blinded and thus at risk of selection bias [35, 37, 38, 40–43]. Out of these studies, three specifically mention this risk. [37, 40, 43]. Only [43] mentions outcome effect sizes. None of the identified RCTs report any conflict of interest.

### Pilot Studies

A total of 11 Pilot Studies were identified in our search [44–54]. These studies were not included in the RCT group because of the lack of a control group, randomization and/or lack of detail of sessions and data. Nevertheless, these pilot studies present significant results in terms of usability and safety. The same analysis procedure used for RCT was performed in these publications, and results are summarized in Tables 5 and 6. A comprehensive list of the identified pilot studies is presented in Additional file 1.

**Table 5:** Pilot Study Experiment Settings

Author	Participants (n,age,sex (men),duration (y), MDS-UPDRS)	Inclusion Criteria	Exclusion Criteria	Aim	Sensor / Platform	Game	Intervention	Control Group?	Setting
Summa et al. (2013) [44]	N: 5 Age (y): 60–76 MDS-UPDRS: Not provided	• PD Diagnosis		Evaluate the feasibility of Kinect-based rehabilitation exercises	Kinect	Custom Game, Whole body movement to reach target	10 sessions, 2 per week, 40 min each Total time: 6.67 h	No	Supervised
Palacios-Navarro et al. (2015) [45]	N: 7 Age (y): 66.8 Sex (men): 3 MDS-UPDRS: Not provided	• MMSE > = 24 • No uncontrolled chronic diseases	• Other neurological diseases • History of falls	Evaluate the feasibility of the proposed scenario	Kinect	Custom game, Mole Hunt with lateral leg movements	4 sessions per week during 5 weeks, 30 min per session Total time: 10 h	No	Supervised by a caregiver
Summa et al. (2015) [46]	N: 7 Age (y): 67 Sex (men): 5 Duration (y): 5 MDS-UPDRS: 15 + − 10	• PD Diagnosis • Ability to stand up and walk a few steps without aid	• Other neurological diseases • Severe receptive aphasia • Inability to perform TUG	Evaluate the improvement in movement speed	Kinect	Custom Game, body movements to reach target	2 sessions per week during 5 weeks, 40 min per session Total time: 6.67 h	No	Supervised
Goncalves et al. (2014) [47]	N: 15 Age(y): 68.70 Sex (men): 7 Duration (y): 7.3 MDS-UPDRS: Not provided	• PD Diagnosis • H&Y Scale II-IV	• Dementia or mental disorders • Changes in medication	Analyze the effect of virtual sensorimotor activity on gait disorders of PD patients	WBB	Wii Fit Plus	2 sessions per week during 7 weeks, 40 min per session Total time: 9,33 h	No	Supervised
Pompeu et al. (2015) [48]	N: 6 Age (y): 61 Sex (men): 4 MDS-UPDRS: 15 (Motor)	• PD Diagnosis • H&Y Scale I-III • MMSE > = 20 • BBS > = 46	• Uncontrolled Hypertension • Heart conditions • Seizures • Orthopedic or neurological diseases • Visual or auditory alterations	Evaluate the use of Kinect Adventures Games on PD Rehabilitation	Kinect	Kinect Adventures Games: SpacePop, 20,000 Leaks, Reflex Ridge and River Rush	3 sessions per week during 4 weeks (total of 14), 60 min per session Total time: 12 h	No	Supervised
Pompeu et al. (2014) [49]	N: 7 Age (y): 72 Sex (men): 6 MDS-UPDRS: 33.6	Same as above (Except H&Y Scale II-III)	Same as above	Assess the feasibility, safety and outcomes of Kinect PD rehabilitation	Kinect	Same as above	Same as above	No	Supervised
Negrini et al. (2017) [50]	N: 27 Age (y): 66 Sex (men): 14	• PD Diagnosis • Able to walk • MMSE > = 26 • Tinetti Balance Scale > 5 • BBS > = 19 • Limitation in activities of daily living	• Cognitive inability to participate • Presence of lower limb problems • History of falls • Cerebrovascular lesions • Other diseases	Evaluate the impact of 10 vs 15 sessions of Wii Fit	WBB	Wii Fit, Wii Balance	2 or 3 sessions per week during 5 weeks, 20 min per session of Wii Fit and 10 min of Wii Balance Total time: 5 h	No	Supervised
Nuic et al. (2018) [51]	N: 10 Age (y): 64.2 Sex (men): 5 MDS-UPDRS: 20.3 (Motor)	• PD Diagnosis • Age < 71 • Presence of FOG • MMSE > = 24	• Inability to walk independently • Other diseases	Determine the feasibility of a custom videogame to treat gait and balance disorders	Kinect	Custom Game, body movements to reach target	3 sessions per week during 6 weeks. Time increases from 15 min (first session) to 40 min (9th session and onwards) Total Time: 11 h (Approximation)	No	Supervised
Cikajlo et al. (2018) [52]	N: 28 Age (y): 68 Sex (men): 12 MDS-UPDRS: 29.54 (Motor)	• PD Diagnosis • H&Y Scale II-III • MMSE > 24	• None specified	Evaluate a Kinect-based telerehabilitation system	Kinect	Custom Game, use arm movements to reach target	Training in clinical setting, followed by 2–3 weeks of 30–60 min sessions	No	Unsupervised (with training)
Pradhan (2018) [53]	N: 3 Age (y): 65.3 Sex (men): 1 MDS-UPDRS: Not provided	• PD Diagnosis • No concurrent neurological disease • Able to walk	• None specified	Evaluate the use of Kinect for PD Rehabilitation	Kinect	Kinect commercial games: Your shape Fitness Evolved, Brain and Body Connection, Kinect Adventures	60 min sessions, with manually-controlled increasing difficulty, for 3 weeks. Total time: Unspecified	No	Supervised
Alves et al. (2018) [54]	N: 27 Age (y): 61 Sex (men): 25 MDS-UPDRS: Not provided	• PD Diagnosis • H&Y Scale I-III • MMSE > = 24 • Stable medication • Good visual/auditory acuity	• Other disorders • Previous experience with Wii or Kinect • Geriatric depression score > 6	Compare the effect of Wii and Kinect in PD rehabilitation	WBB, Kinect	Wii Fit Plus Kinect Adventures Your Shape: Fitness Evolved	2 sessions per week during 5 weeks, 60 min per session Total time: 10 h	Yes	Supervised

**Table 6:** Pilot Study Experiment Outcomes. BES = Balance Evaluation System, FIM = Functional Independence Measure, nMWT = n Minutes Walking Test. S&E = Schwab and England Independence Scale. Results are presented as mean (standard deviation) unless stated otherwise. Outcomes are adimensional where no units are stated

Author	Outcome	Baseline (Mean (SD))	Post Intervention (Mean (SD))	Main Results	Main conclusion
Summa et al. (2013) [44]	Not described	Not described	Not described	MWT and TUG improvement in 3/5 users Increase in movement acceleration	Statistically significant improvement of patients.
Palacios-Navarro et al. (2015) [45]	10MWT (s)	• 12 (6)	• 10 (5)	Improvement in 10MWT test, statistically significant (*p* = 0.002)	Scenario is feasible, but long term impact unknown. Adaption to home scenario proposed.
Summa et al. (2015) [46]	TUG (s) 10MWT (s)	• 15 (12) • 12 (12)	• 16 (15) • 12 (13)	No significant changes on standard outcomes. Improvement in absolute average acceleration	Scenario appears safe to use, possible training-induced reduction of bradykinesia.
Goncalves et al. (2014) [47]	UPDRS Motor S&E Scale FIM	• 28.5 (9.91) • 79.3 (9.61) • 114.3 (6.07)	• 15.8 (7.49) • 90.0 (6.54) • 121.3 (2.65)	Statistically significant differences for all outcomes (*p* < 0.001) Increase in stride length and gait speed	WBB gait motor training is effective, even in a short time period.
Pompeu et al. (2015) [48]	Limit of Stability	• 118.5 (28.0)	• 163.7 (38.3)	Improvement in limit of stability, statistically significant (*p* < 0.05)	Kinect training is safe and promotes improvement in postural control.
Pompeu et al. (2014) [49]	6MWT (m) PDQ-39 BES	• 399.3 (72.4) • 27.8 (8.3) • 74.1 (12.7)	• 429.5 (90.6) • 22.34 (1.9) • 88.9 (14.8)	Effect sizes of 0.3 for 6MWT, 0.7 for PDQ-39 and 1.1 for BES. Positive outcome, but the sample size does not provide significant results	Training with Kinect is safe and feasible. Cardiopulmonary endurance, balance, gait and quality of life improves
Negrini et al. (2017) [50]	BBS Tinetti balance scale Tinetti gait scale	10 sessions: • 46.6 (5.8) • 11.9 (3.2) • 8.1 (2.9) 15 sessions: • 40.1 (7.6) • 12.2 (3.0) • 9.0 (1.8)	10 sessions: • 51.3 (7.2) • 14.5 (3.2) • 10.4 (1.8) 15 sessions: • 46.3 (7.1) • 13.6 (3.1) • 10.1 (2.2)	All results statistically significant within groups (p < 0.001). 10 sessions suffice to see results. No significantly different results with the 15 sessions group	Wii Fit is cost-efficient and provides result, home scenario may be viable.
Nuic et al. (2018) [51]	UPDRS (Motor) GABS Scale FOG Questionnaire	• 20.3 (7.8) • Not reported • 25.9 (5.4)	• Not Reported • −38 points across all users • − 39 points across all users	Statistically significant improvement in FOG and GABS (*p* < 0.02), differences in UPDRS scores not significant (*p* = 0.13).	Game is feasible, well accepted and shows potential for PD rehabilitation.
Cikajlo et al. (2018) [52]	UPDRS (Motor) Nine-Hole Test Box and Blocks Test	• 29.54 (10.33) • 28.01 (6.59) • 47.27 (10.68)	• 27.29 (10.38) • 26.48 (7.30) • 51.65 (11.26)	Statistically significant (*p* < 0.003) improvement in UPDRS and Box and Blocks, nonsignificant in Nine-Hole Test (*p* = 0.089)	Telerehabilitation is possible with training and remote supervision, and achieving significant clinical outcomes is possible-
Pradhan (2018) [53]	Functional Reach Test (cm) 6MWT (m) Gait speed (m/s)	• 25.65(5.92) • 502.11(36.54) • 7.1(0.6)	• 33.71(2.84) • 560.53(23.83) • 6.97(0.90)	Statistically significant differences (p < 0.05) on functional reach test	Certain improvements observed
Alves et al. (2018) [54]	TUG (s) 10 MWT (s) 10 MWT (m)	• 10.44(2.16)/11.68(5.22) • 7.03(1.52)/7.07(1.40) • 1.47(0.31)/1.44(0.21) (Wii / Xbox)	• 9.77(1.5)/9.82 (3.41) • 6.89(1.05)/6.96(1.46) • 1.47(0.23)/1.48(0.27) (Wii / Xbox)	Statistically significant improvement in Wii outcomes (*p* > 0.049), improvement in Kinect group non-significant, no improvement in control group.	Wii seems to perform better than Kinect, since only the first group shows statistically significant improvement.

Pilot studies are usually focused on proving the feasibility of rehabilitation scenarios, in most cases regarding its safety as well as ease of use in PD patients. Interestingly, most studies reported improvement, and none reported deterioration or safety risk as a consequence of the study. However, due to low sample sizes and insufficient intervention durations, outcome changes were not statistically significant in some cases. As it is the case for the RCTs, the evaluated scenarios were a mixture of custom and commercial games, with both of these providing similar results. Two of the pilot studies [45, 50] mention the possibility of adapting their modules to a minimally supervised home scenario, a level of supervision that could be achieved, for example, by relatives. The question of therapy adherence is mentioned, but, as mentioned in the previous section, challenges regarding safety remain largely unaddressed.

We also discovered a very recent study offering the first comparison between exergaming platforms specifically for PD rehabilitation, specifically WBB and Kinect [54]. Outcomes of this study suggest the WBB may be more adequate for PD rehabilitation, at least in the disease factors that can be evaluated through the 10MWT or TUG tests. This aspect is further mentioned in the discussion.

## Discussion

The research activity in the area of exergames for the rehabilitation of PD patients has improved impressively in recent years. The previous systematic review by Barry et al. [34] could only analyze seven studies and just one of these qualified as RCT. By turn, in this systematic review, we included 64 new studies, eight of which qualified as a RCT. Our findings indicate that the putative improvement in motor skills suggested before 2014 has been confirmed by several independent studies including both, RCTs and pilot studies. As a whole, from a total of 19 studies where exergame therapy was evaluated (both RCTs and pilot studies), 17 demonstrated improvements in the measured outcomes (8 out of 9 RCTs and 9 out of 11 pilot studies). Additionally, in 7 out of 9 RCTs the intervention group provided better results than the control group in motor outcomes. Finally, none of the studies showed exergames were in any way worse than traditional rehabilitation therapy. Additionally, exergames seem to improve cognitive skills (MOCA scores, attention) in the aforementioned RCT study populations (PD patients with MMSE scores > 24) as well.

From our analysis on recent RCTs, we can conclude that the focus on using the WBB is switching towards the Microsoft Kinect v2, which apparently provides similar results while being easier to use since patients do not have to stand on an elevated platform. Recent research shows how this sensor can provide relevant and accurate gait parameters which can be potentially used to monitor PD patients remotely during rehabilitation sessions. However, RCTs using this platform remain scarce, while positive WBB results are more numerous. Up to this point, all RCTs include an MMSE score of 24 or higher as an exclusion criterion. Thus, the effect of patients with lower MMSE scores is yet to be determined. Considering recent studies show how exergaming does improve cognitive capabilities in PD patients [35], it seems necessary to extend these studies into PD-MCI as well as initial PDD, and evaluate the feasibility of game-based therapy on these patients as well.

An additional advantage presented by the Kinect over the WBB or Wiimote seems to be that the risk of patient not liking the input device used in the game is lower [55]. How PD patients will react to speech control or an improved version of a WBB is unknown, and the number of RCTs involving the Kinect is up to this point insufficient. Future research should also consider new sensors and methods, such as the Leap Motion finger tracking sensor (see Table 7), which, as with the Kinect sensor, does not require the user to wear it or carry any weight. We believe this tendency will continue towards other sensors and scenarios where the user can perform natural movements, such as fine motor training with the Leap Motion sensor.

**Table 7:** Technical Information about the sensors mentioned in this publication

Sensor	Manufacturer	Size (L x W x H cm)	Weight (g)	Way of use	Approximate Cost (Eur)
WBB	Nintendo	49,5 × 30,5 × 5,1	3500	Foot Movements through pressure sensors	70
Wiimote	Nintendo	16 × 3,62 × 3,08	90 (without batteries)	Arm movements through inertial measurement units	38
Kinect	Microsoft	27,94 × 7,11 × 6,6	560	Bodily movements	130
Leap Motion	Leap Motion Inc	8 × 3 × 1,13	32	Finger movements	70

Regarding outcome comparisons between exergaming platforms. A recent pilot study [54] suggests the WBB may be more optimal for Kinect in PD rehabilitation, based on a comparison performed between these two platforms. However, the meta-analysis of RCT outcomes (Table 4) suggests otherwise: We found higher improvements in the TUG test using WBB (1 WBB-Based RCT vs. 1 Kinect-based RCT) and better results with Kinect in BBS (2 WBB-based RCTs vs 2 Kinect-based RCTs).

Considering technical papers, 19 [15–33] out of the 30 [15–33, 55–65] publications in this group focused towards linking sensor parameters to disease severity indicators. The use of these sensors for screening is suggested [22, 33]. The limitations of the current generation of sensors, particularly in fine motorics, is also discussed [29]. In this case, a more dedicated sensor, such as a data glove [56], or the Leap Motion sensor [65] may be used. In addition, there is also clear interest in developing rehabilitation methods that may be performed in an unsupervised home scenario [56, 59–63]. Patient preferences regarding sensors are rarely discussed [55].

In parallel to these findings, 15 meta-analyses and systematic reviews were found [66–80]. These relate to a wide array of topics, however, none of the studies we found related to the specific topic of exergame-based rehabilitation for PD specifically.

This study presents some limitations. Firstly, the lack of standardized outcomes in this research area makes it still difficult to determine which exergaming platform, and method, works best for the specific needs of PD patients. The meta-analysis we could perform in this aspect was limited to the studies presented in Table 4 and, although illustrative, requires significant extension before conclusions may be extracted. It is also early to quantitatively determine the effect of exergame therapy in PD-MCI and initial PDD patients, either in improvement or slowdown of deterioration. Given that the effectiveness of exergames, at least in comparison with traditional rehabilitation, seems to be comparable, it is critical that future RCTs establish the use of a reduced number of standard outcomes such as MDS-UPDRS, BBS or TUG in order to start providing a more significant insight into what scenarios and sensors are actually more effective to train which difficulties of PD patients in particular or motor afflictions in general. If authors adhere to these principles, a repetition of this exercise performed another 5 years from now may very well provide critical insight in the development of game-based PD-specific rehabilitation: Which sensors to use, what game environments are optimal, and which are the ideal difficulty adaptation curves based on UPDRS or MMSE assessments.

Considering the effect of RCTs in cognition, performing a follow-up analysis is also essential to determine long-term patient improvement in all outcomes, especially considering follow-up results seem to be good for cognitive skills, but not for balance.

Although it is still difficult to determine, it seems there is not a big difference between PD-specific games and custom-developed games. For the patients included in the analyzed clinical studies, that is, patients with MMSE scores over 24, and although it is too early to make clear conclusions, it seems that cognitive specific therapy and commercial games have no significant difference in cognitive outcomes, as seen on Table 3, although whether this also applies to PD-MCI patients remains to be determined. An RCT mentions the importance of task specificity in this case [41]. Other studies support the idea that therapy should adapt to the patient’s skills, and this would be possible with custom-developed adaptive exergames, but not with commercial ones.

Regarding sensors, it is clearly essential to further determine what constitutes the right array of sensors to ensure a pleasant experience while the most amount of medically relevant information is captured in the background, adapting to the patients’ skills where possible. Unfortunately, the number of studies in this regard is very limited so far.

Another research field, parallel to the one presented in this work, is the use of Virtual Reality in rehabilitation in general and for PD in particular. A recent systematic review [81] summarizes recent advances in this area in the last years. Similarly to exergame-based rehabilitation, limited evidence shows positive effects, but additional studies are required to confirm the results.

## Conclusions

In our opinion, the main conclusion of this study is that exergame PD rehabilitation is indeed safe, feasible and effective, and in some cases [37, 38, 40, 42] even more effective than traditional rehabilitation and regular exercise. Exergames also provide the possibility of a home rehabilitation and remote monitoring scenario, which are limitations of traditional rehabilitation methods. There is contradictory evidence regarding which exergame platform performs better at this point.

Regarding future work, in addition to providing further, high-quality clinical trials in order to confirm the hypotheses so far presented, studies should focus on adapting existing therapies to home scenarios, adding further data collection procedures, such as monitoring hand tremors, perhaps with a finger sensor [82]. Additional variables such as dysphonia [83] and heart rate variability [84] may also be of interest. Evaluating the feasibility of expanding PD Exergame therapy to patients with PD-MCI and PDD, and including new interaction techniques such as fine motoric training and speech control are also points of interest. Finally, programs should be tailored to patient characteristics, both physical and cognitive.

## Additional file

Additional file 1: **Table S1.** Overview of the selected RCTs and Pilot Studies (PDF 276 kb)
