# Supplementary material for: Recent advances in rehabilitation for Parkinson’s Disease with Exergames: A Systematic Review
Source: J Neuroeng Rehabil. 2019 Jan 29;16:17. doi: 10.1186/s12984-019-0492-1 (PMC6352377; doi:10.1186/s12984-019-0492-1)
Supplement: Supplementary file 1 — Table S1. Overview of the selected RCTs and Pilot Studies (PDF 276 kb) [file 12984_2019_492_MOESM1_ESM.pdf]

Supplementary Table 1: Overview of the selected RCTs and Pilot Studies

Clinical Trials	Authors	Title	Journal
Pompeu et al. (2012) [35]	J. E. Pompeu, F. A. dos Santos Mendes, K. G. da Silva, A. M. Lobo, T. de Paula Oliveira, A. P. Zomignani, *et al.*	Effect of Nintendo Wii™-based motor and cognitive training on activities of daily living in patients with Parkinson's disease: A randomised clinical trial.	*Physiotherapy,* vol. 98, pp. 196-204, 2012.
Allen et al. (2017) [36]	N. E. Allen, J. Song, S. S. Paul, S. Smith, J. O'Duffy, M. Schmidt, *et al.*	An interactive videogame for arm and hand exercise in people with Parkinson's disease: A randomized controlled trial.	*Parkinsonism & Related Disorders, 2017.*
Liao et al. (2015) [37]	Y.-Y. Liao, Y.-R. Yang, S.-J. Cheng, Y.-R. Wu, J.-L. Fuh, and R.-Y. Wang.	Virtual reality–based training to improve obstacle-crossing performance and dynamic balance in patients with Parkinson's disease.	*Neurorehabilitation and neural repair,* vol. 29, pp. 658-667, 2015.
Shih et al. (2016) [38]	M.-C. Shih, R.-Y. Wang, S.-J. Cheng, and Y.-R. Yang.	Effects of a balance-based exergaming intervention using the Kinect sensor on posture stability in individuals with Parkinson's disease: a single-blinded randomized controlled trial.	*Journal of neuroengineering and rehabilitation,* vol. 13, p. 78, 2016.
Ribas et al. (2017) [39]	C. G. Ribas, L. A. da Silva, M. R. Corrêa, H. G. Teive, and S. Valderramas.	Effectiveness of exergaming in improving functional balance, fatigue and quality of life in Parkinson's disease: A pilot randomized controlled trial.	*Parkinsonism & Related Disorders,* 2017.
Zimmermann et al. (2014) [40]	R. Zimmermann, U. Gschwandtner, N. Benz, F. Hatz, C. Schindler, E. Taub*, et al.*	Cognitive training in Parkinson disease Cognition-specific vs nonspecific computer training .	*Neurology,* vol. 82, pp. 1219-1226, 2014.
Song et al. (2017) [41]	J. Song, S. S. Paul, M. J. D. Caetano, S. Smith, L. E. Dibble, R. Love*, et al.*	Home-based step training using videogame technology in people with Parkinson's disease: a single-blinded randomised controlled trial.	*Clinical rehabilitation,* p. 0269215517721593, 2017.
Ferraz et al. (2018) [42]	D. D. Ferraz, K. V. Trippo, G. P. Duarte, M. G. Neto, K. O. B. Santos, and J. Oliveira Filho.	The effects of functional training, bicycle exercise and exergaming on walking capacity of elderly with Parkinson's disease: a pilot randomized controlled single-blinded trial.	*Archives of physical medicine and rehabilitation,* 2018.
Tollar et al. (2018) [43]	J. Tollar, F. Nagy and T. Hortobagyi	Vastly different exercise programs similarly improve parkinsonian symptoms: A randomized clinical trial	*Gerontology, pp. 1-8, 2018*
**Pilot Studies**	**Authors**	**Title**	**Journal**
Summa et al. (2013) [44]	S.Summa,A.Basteris,E.Betti,and V.Sanguineti	A feasibility study on using kinect™ for the rehabilitation in persons with Parkinson's disease.	*Gait & Posture,* vol. 37, p. S15, 2013.
Palacios-Navarro et al. (2015) [45]	G. Palacios-Navarro, I. García-Magariño, and P. Ramos-Lorente,	A Kinect-based system for lower limb rehabilitation in Parkinson's disease patients: a pilot study.	*Journal of medical systems,* vol. 39, p. 103, 2015.
Summa et al. (2015) [46]	S. Summa, A. Basteris, E. Betti, and V. Sanguineti,	Adaptive training with full-body movements to reduce bradykinesia in persons with Parkinson's disease: a pilot study.	*Journal of neuroengineering and rehabilitation,* vol. 12, p. 16, 2015.
Goncalves et al. (2014) [47]	G. B. Gonçalves, M. A. A. Leite, M. Orsini, and J. S. Pereira,	Effects of using the nintendo wii fit plus platform in the sensorimotor training of gait disorders.	Parkinson's disease," *Neurology international,* vol. 6, 2014.
Pompeu et al. (2015) [48]	J. E. Pompeu, C. Torriani-Pasin, F. Doná, F. F. Ganança, K. G. da Silva, and H. B. Ferraz,	Effect of Kinect games in postural control of patients with Parkinson's disease.	*Proceedings of the 3rd 2015 Workshop on ICTs for improving Patients Rehabilitation Research Techniques*, 2015, pp. 54-57.
Pompeu et al. (2014) [49]	J. Pompeu, L. Arduini, A. Botelho, M. Fonseca, S. A. A. Pompeu, C. Torriani-Pasin*, et al.*,	Feasibility, safety and outcomes of playing Kinect Adventures!™ for people with Parkinson's disease: a pilot study.	*Physiotherapy,* vol. 100, pp. 162-168, 2014.
Negrini et al. (2017) [50]	S. Negrini, L. Bissolotti, A. Ferraris, F. Noro, M. D. Bishop, and J. H. Villafañe	Nintendo Wii Fit for balance rehabilitation in patients with Parkinson's disease: A comparative study.	*Journal of Bodywork and Movement Therapies, vol. 21, pp. 117-123, 2017.*
Nuic et al. (2018) [51]	D. Nuic, M. Vinti, C. Karachi, P. Foulon, A. Van Hamme and M.-L. Welter	The feasibility and positive effects of a customised videogame rehabilitation programme for freezing of gait and falls in Parkinson's Disease patients: A	*Journal of Neuroengineering and Rehabilitation, vol. 15, p.31, 2018*

		pilot study	
Cikajlo et al. (2018) [52]	Cikajlo, A. Hukic, I. Dolinsek, D. Zajc, M. Vesel, T.Krizmanik, B. Blazica, A. Biasizzo, F. Novak and K. P. Potisk	Can telerehabilitation games lead to functional improvement of upper extremities in individuals with Parkison's Disease?	*International Journal of Rehabilitation Research, vol. 41, p.230, 2018*
Pradhan (2018) [53]	S. Pradhan	The use of commercially available games for a combined physical and cognitive challenge during exercise for individuals with Parkinson's Disease – a case series report"	*Physiotherapy theory and practice, pp. 1-8, 2018*
Alves et al. (2018) [54]	M.L. Alves, B.S. Mesquita, W.S. Morais, J.C. Leal, C.E. Satler and F.A. dos Santos Mendes	Nintendo Wii versus Xbox Kienct for assisting people with Parkinson's Disease	*Perceptual and motor skills, vol. 125, pp. 546-565, 2018*
